# Haemotropic mycoplasmas: What's their real significance in cats?

**DOI:** 10.1016/j.jfms.2010.03.011

**Published:** 2010-05

**Authors:** Séverine Tasker

**Affiliations:** Senior Lecturer in Small Animal Medicine, The Feline Centre, School of Clinical Veterinary Science & Langford Veterinary Services, University of Bristol, Langford, Bristol BS40 5DU, UK

## Abstract

**Practical relevance:**

The feline haemotropic mycoplasmas (‘haemoplasmas') are a group of bacteria that can induce haemolytic anaemia in cats. *Mycoplasma haemofelis* is the most pathogenic of the species; ‘*Candidatus* Mycoplasma haemominutum’ and ‘*Candidatus* Mycoplasma turicensis’ are less pathogenic. The natural route of transmission of feline haemoplasma infection has not been confirmed, but fleas are implicated. When disease results, common clinical signs are pallor, lethargy, anorexia, weight loss, depression, dehydration and pyrexia. Treatment with tetracyclines or fluoroquinolones is usually effective at resolving clinical disease, but clearance of infection may not result.

**Global importance:**

The feline haemoplasmas are found worldwide, although prevalence varies geographically.

**Patient group:**

Older male non-pedigree cats are believed to be at increased risk of haemoplasma infection, although younger cats are possibly more likely to show clinical disease associated with *M haemofelis*.

**Clinical challenges:**

The significance of feline haemoplasma infection is difficult to determine due to the existence of asymptomatic carrier cats and the variable pathogenicity of the haemoplasma species. Polymerase chain reaction (PCR) results should be interpreted in the light of the patient's clinical signs and haematological findings, infecting haemoplasma species and level of haemoplasma DNA present in the blood. Trial antibiotic treatment for haemoplasmosis may be warranted in suspected cases while awaiting PCR results.

**Evidence base:**

Aspects of feline haemoplasmosis, particularly risk factors, pathogenesis, diagnostic methods and treatment, have been the focus of much recent research. This article draws on the current evidence base with a view to helping clinicians diagnose and manage cases more effectively.
